# Computed tomography imaging of resuscitative endovascular balloon occlusion of the aorta (REBOA): pearls and pitfalls

**DOI:** 10.1007/s11604-021-01166-w

**Published:** 2021-07-03

**Authors:** Ryo Aoki, Yusuke Kobayashi, Shintaro Nawata, Hiroyuki Kamide, Toh Yamamoto, Shintaro Furugori, Zenjiro Sekikawa, Daisuke Utsunomiya

**Affiliations:** 1 Diagnostic Radiology, Yokohama City University Graduate School of Medicine, Yokohama-shi, 4-57 Urafunecho, Minami-ku, Yokohama-shi, Kanagawa, 232-0024 Japan; 2 Diagnostic Radiology, Yokohama City University Medical Center, Yokohama-shi, Kanagawa, Japan; 3 Advanced Critical Care and Emergency Center, Yokohama City University Graduate School of Medicine, Yokohama-shi, Kanagawa, Japan

**Keywords:** Resuscitative endovascular balloon occlusion of the aorta, Computed tomography, Complications, Pitfalls

## Abstract

Resuscitative endovascular balloon occlusion of the aorta (REBOA) is performed in patients with hemorrhagic shock who develop massive subdiaphragmatic bleeding. This procedure enables rapid and less invasive aortic blockade compared to resuscitative thoracotomy and aortic cross-clamp procedures. However, the REBOA procedure is often blindly performed in the emergency department without fluoroscopy, and the appropriateness of the procedure may be evaluated on computed tomography (CT) after REBOA. Therefore, radiologists should be familiar with the imaging features of REBOA. We present a pictorial review of the radiological findings of REBOA along with a description of the procedure, its complications, and pitfalls.

## Introduction

High-energy trauma is defined as open or closed injuries caused by forces (e.g., motor vehicle accidents). Organs and tissues can sustain extensive damage due to a high amount of kinetic energy, causing severe bleeding. In such time-sensitive and life-threatening situations, urgent endovascular techniques are essential [1].

Resuscitative endovascular balloon occlusion of the aorta (REBOA) is a resuscitation procedure in which a balloon catheter is inserted into the aorta to create a balloon blockade that increases proximal arterial pressure to maintain central organ perfusion while controlling distal subdiaphragmatic hemorrhage [2]. Aortic blockade with REBOA is less invasive than resuscitative thoracotomy and aortic cross-clamp procedures [3]. Ideally, the REBOA procedure is performed under guidance by fluoroscopy and/or ultrasound for appropriate placement without complications. However, the REBOA procedure is highly emergent and may be performed blindly because these modalities and human resources are often unavailable in emergency situations. Furthermore, the assessment by ultrasonography is operator-dependent. On the other hand, computed tomography (CT) is advantageous because CT provides objective assessment, information on REBOA placement, and status of various organs. Therefore, radiologists should be familiar with the imaging features of REBOA. We present a pictorial review of the radiological findings of REBOA along with a description of its procedure, classification, complications, and pitfalls.

## REBOA procedure

The procedure was performed using a 7-French REBOA device (Rescue Balloon^™^ or Rescue Balloon ER™, Tokai Medical Products Corp., Kasugai, Aichi, Japan) according to the following steps: (1) establish arterial access (usually through the common femoral artery considering ultrasound guidance or cut-down approach) and place the sheath, (2) insert the REBOA catheter (balloon catheter) over the guidewire, (3) remove the guidewire and insert the stylet into the REBOA catheter to avoid kinking or migration of the balloon catheter because of the arterial pressure during balloon inflation, and 4) inflate the balloon. Ideally, balloon proximal and distal arterial pressure monitoring lines (e.g., left radial and femoral artery) allow us to determine adequate balloon positioning and inflation [4].

## Types of REBOA based on balloon inflation

A trade-off between the occlusion duration of REBOA and tissue ischemia exists. There are three types of balloon inflation techniques. The REBOA type is chosen according to the disease severity. To reduce the risk of tissue ischemia, partial or intermittent REBOA is performed [5, 6].

- ***Complete REBOA***
  Balloon inflation can result in the cessation of distal pulse pressure by total aortic occlusion. However, complete REBOA increases the risk of distal ischemia and cardiac afterload.
- ***Partial REBOA***
  The balloon is partially inflated, and the aorta is not completely occluded. However, the control of downstream bleeding may be incomplete.
- ***Intermittent REBOA***
  Repeated inflation and deflation of the balloon are performed to restore downstream perfusion. Intermittent REBOA is expected to improve survival while minimizing ischemia–reperfusion injury [6].

## Balloon positions and indications

**Fig. 1:**
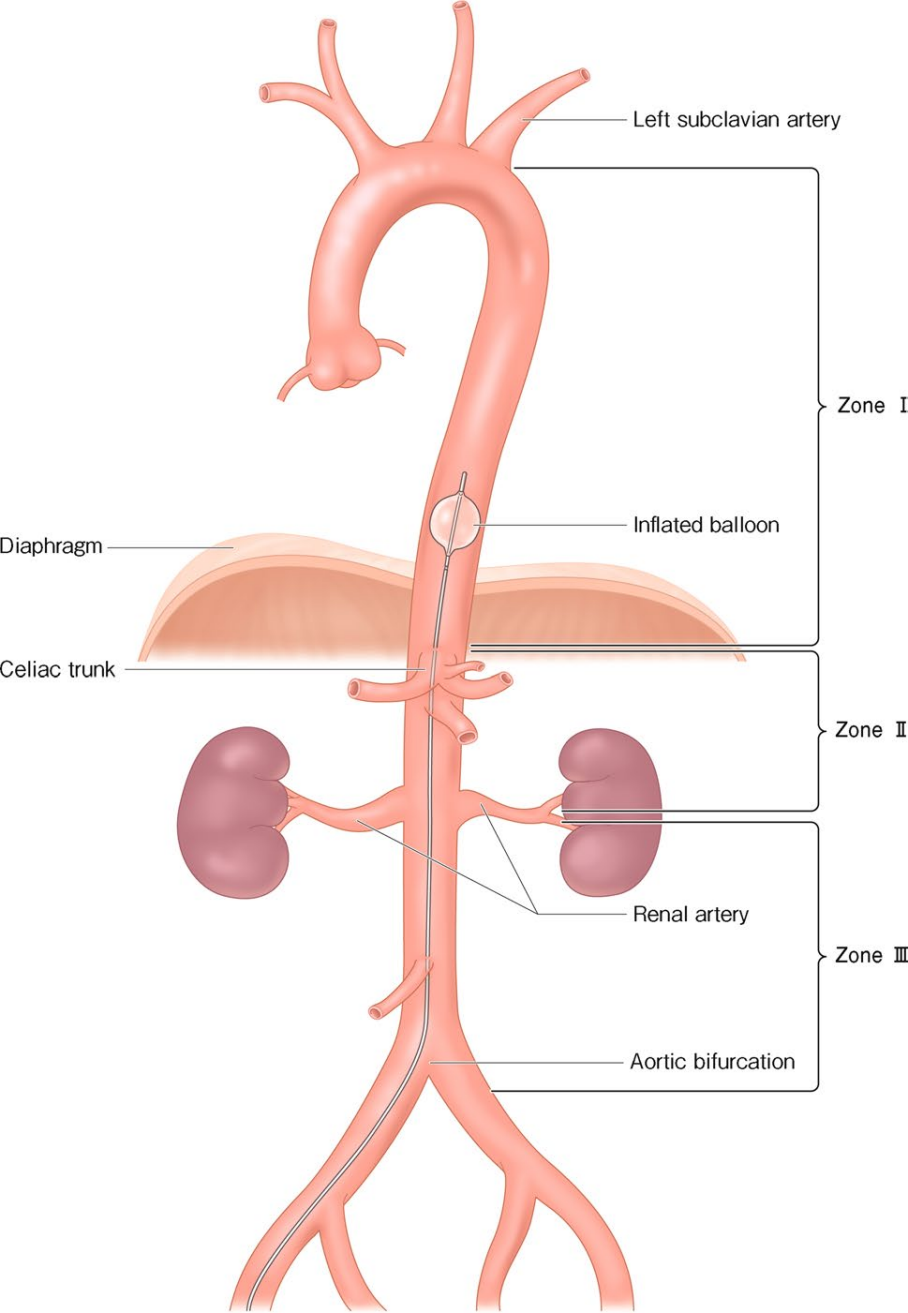
The balloon inflation site is divided into three zones. Zone I is the area between the origin of the left subclavian artery and the celiac artery. Zone II is defined as the area between the lower end of Zone I and the top of Zone III. Zone III is the area between the origin of the lowest renal artery and the aortic bifurcation

**Fig. 2:**
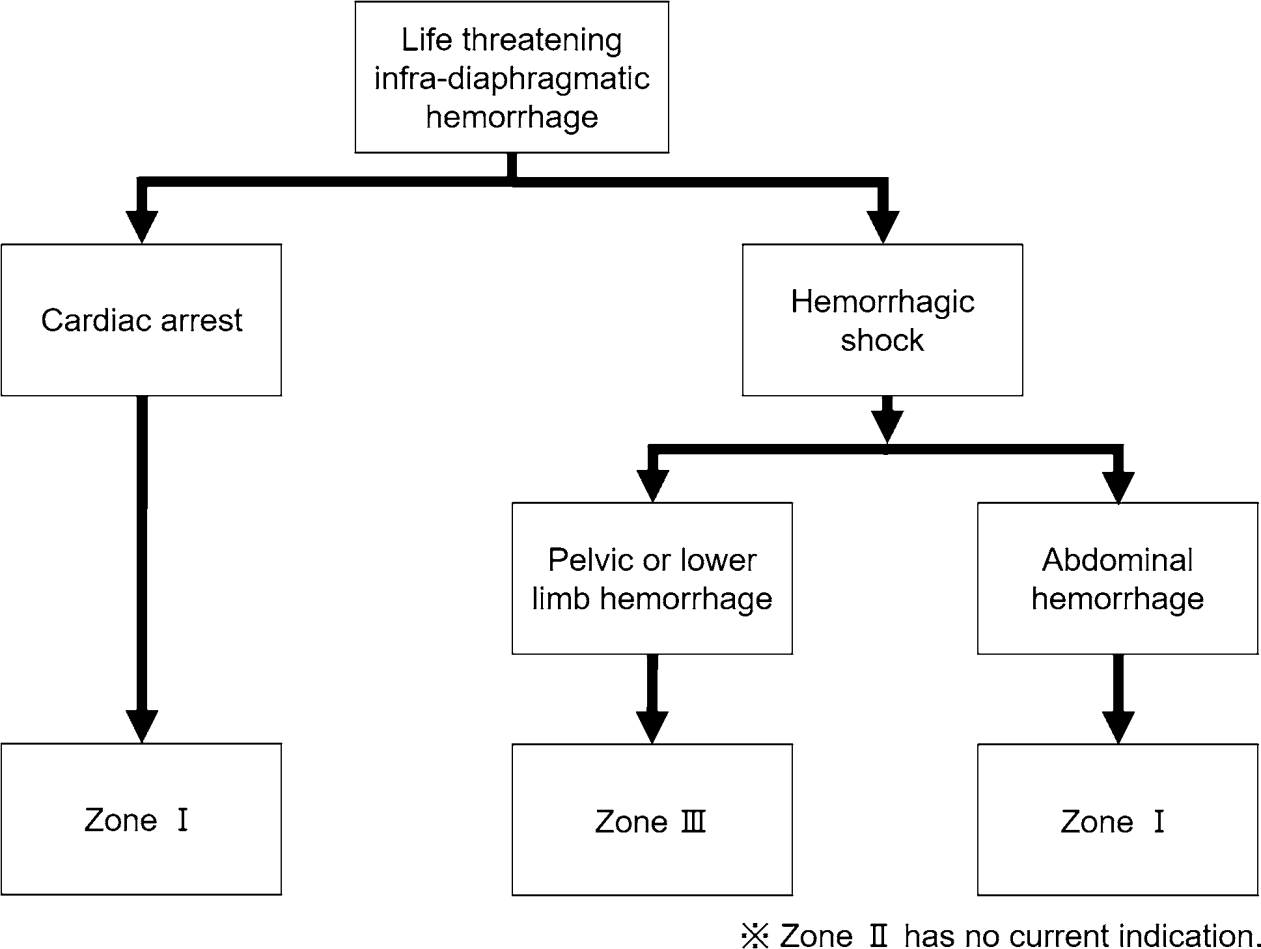
Algorithm showing the appropriate Zone for resuscitative endovascular balloon occlusion of the aorta

The balloon position is divided into three zones (Figs.  1, 2) [7].

### Zone I

The balloon is positioned in the aorta between the origin of the left subclavian and celiac arteries. Zone I is indicated in cardiac arrest or hemorrhagic shock with intra-abdominal hemorrhage (e.g., severe trauma and rupture of an abdominal aortic aneurysm).

### Zone II

Zone II is defined as the area between Zones I and III. Placement in Zone II should be contraindicated due to risk of gastrointestinal ischemia.

### Zone III

The balloon is positioned in the aorta between the origins of lowest renal artery to the aortic bifurcation. Zone III is indicated in patients with life-threatening pelvic or lower limb hemorrhage (e.g., pelvic fracture and postpartum hemorrhage).

## Evaluation of CT after REBOA

**Fig. 3:**
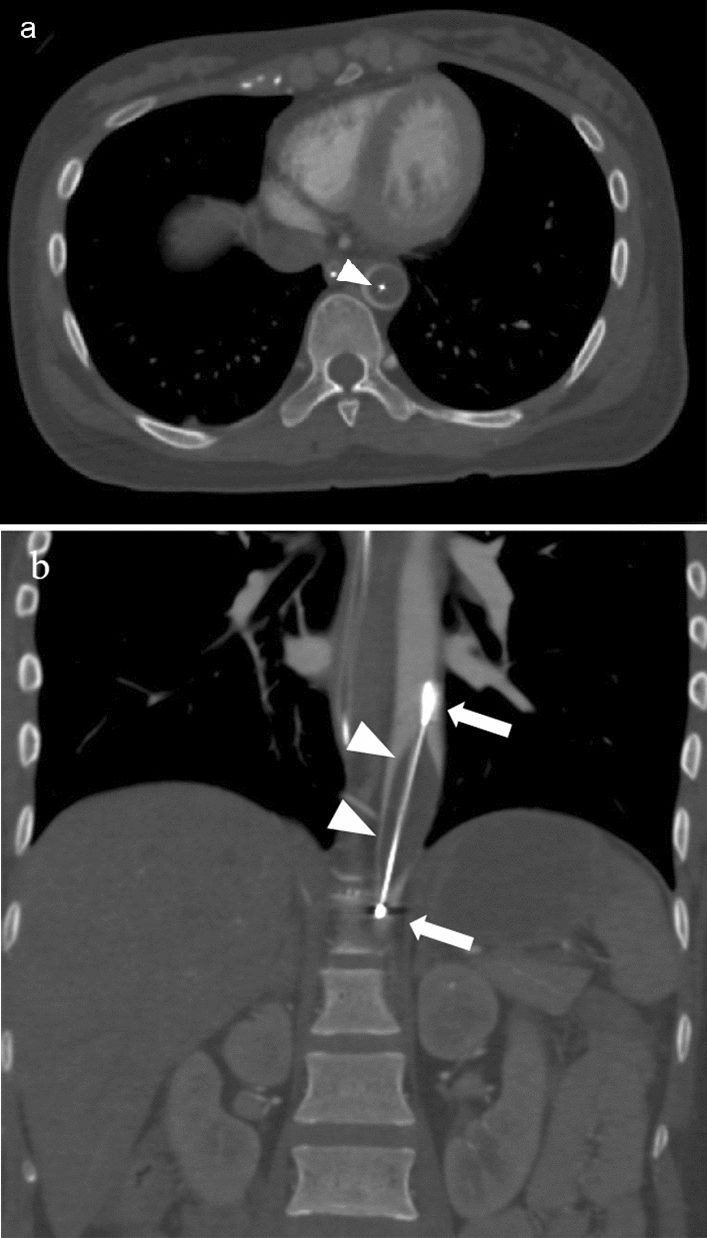
Contrast-enhancement CT (**a**: axial, **b**: coronal view) shows an appropriate partial resuscitative endovascular balloon occlusion of the aorta placed in Zone I. The white arrows represent radiopaque markers of the balloon catheter, and the white arrowheads represent the stylet in the balloon catheter

**Fig. 4:**
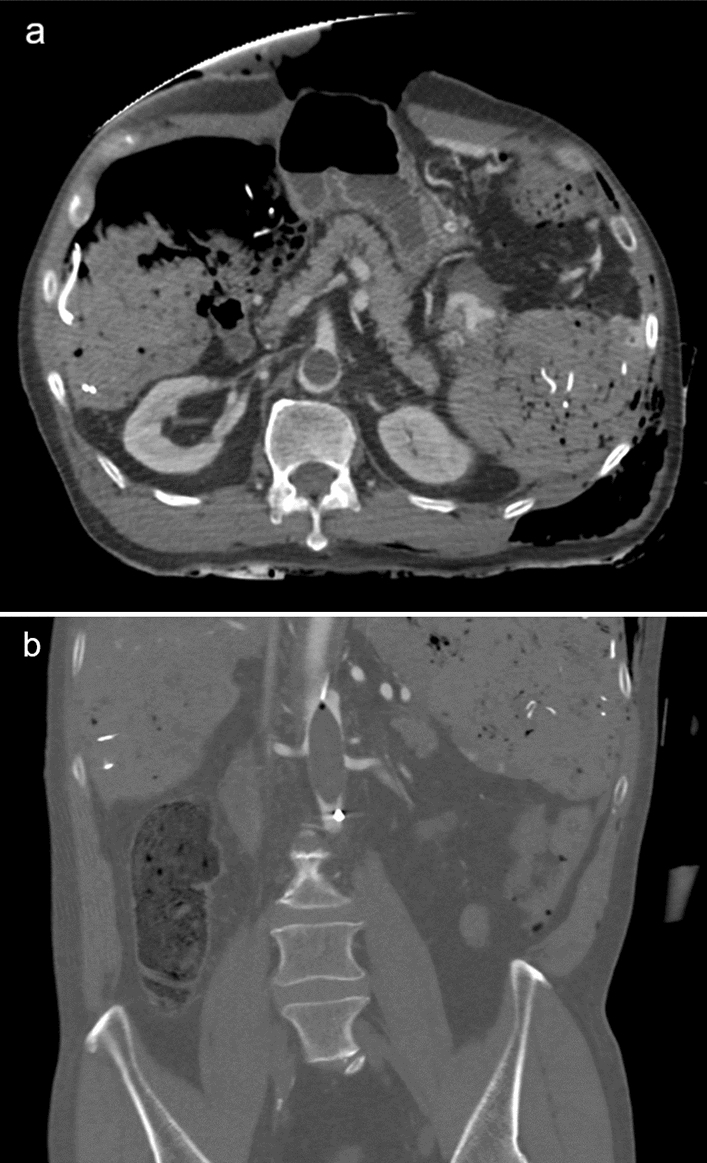
Contrast-enhancement computed tomography shows an inappropriate resuscitative endovascular balloon occlusion of the aorta for a patient with multiple trauma (**a**: axial, **b**: coronal view). The balloon was positioned at Zone II, and the stylet was not inserted in the balloon catheter

Radiologists must assess the appropriateness of REBOA procedure (Figs.  3, 4) and detect active bleeding on CT. In some patients, distal perfusion and active extravasation can still be detected in the setting of complete REBOA. It is speculated that the balloon and aortic wall were not watertight [8] with multiple collateral pathways from proximal to distal aorta [9].

## Complications

There are two main types of complications: perfusion-related and procedure-related complications.

### Perfusion-related complications

Perfusion-related complications include distal ischemia–reperfusion injury and exacerbation of proximal bleeding.

#### Organ ischemia and reperfusion injury

**Fig. 5:**
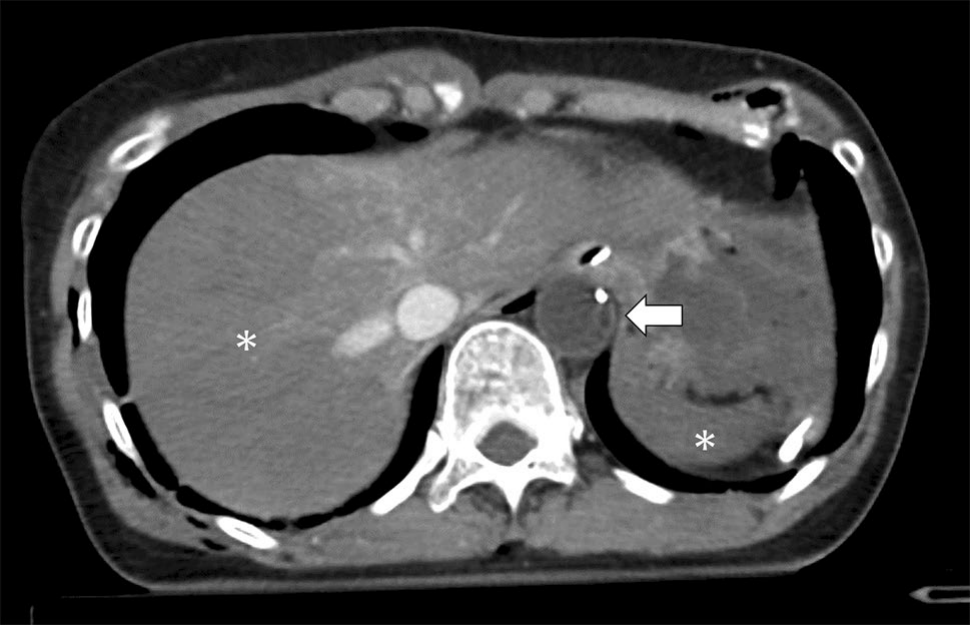
Abdominal contrast-enhancement computed tomography (venous phase) with complete Zone I resuscitative endovascular balloon occlusion of the aorta (arrow) showing hypo-enhancement (hypoperfusion) in the liver and spleen (asterisks)

REBOA can control subdiaphragmatic hemorrhage by decreasing distal arterial pressure; however, it may decrease organ perfusion (Fig.  5). Ischemia and reperfusion injury are common [10]; thus, inflation time should be minimized to prevent irreversible ischemic organ injury. Ideally, total aortic occlusion time < 30 min avoids ischemic complications [11].

#### Exacerbation of bleeding

**Fig. 6:**
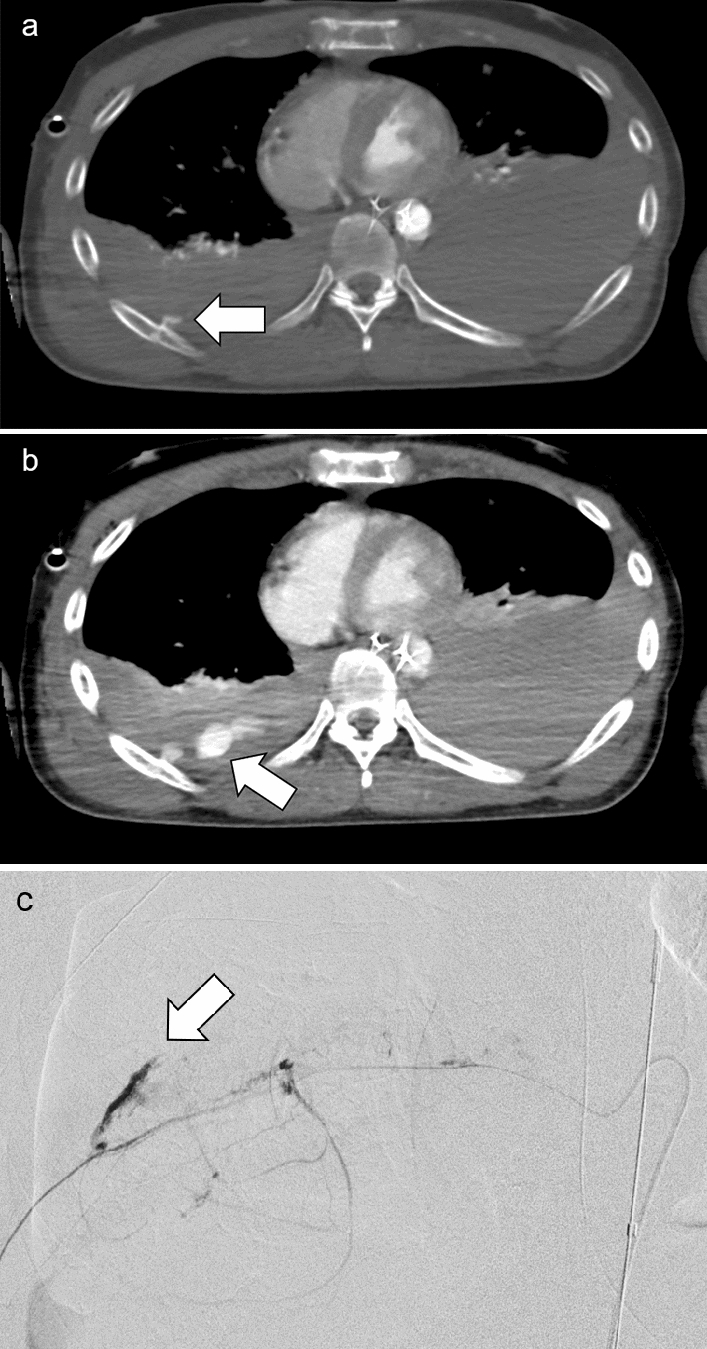
Thoracic contrast-enhancement computed tomography (**a**: arterial phase, **b**: venous phase) with Zone I resuscitative endovascular balloon occlusion of the aorta under temporally deflated status after total inflation in the patient with multiple traumas showing extravasation (**a** and **b**, arrow) from the right intercostal artery, which was confirmed by angiography (**c**, arrow). The bleeding was treated by transcatheter arterial embolization

REBOA can worsen hemorrhage above the diaphragm [12] because of increased arterial pressure; therefore, the necessity of REBOA should be carefully evaluated (Fig.  6).

### REBOA-placement-related complications

REBOA-placement-related complications include iatrogenic vessel injuries and inappropriate device insertion (e.g., kinking, loop formation, and migration).

#### Loop formation

**Fig. 7:**
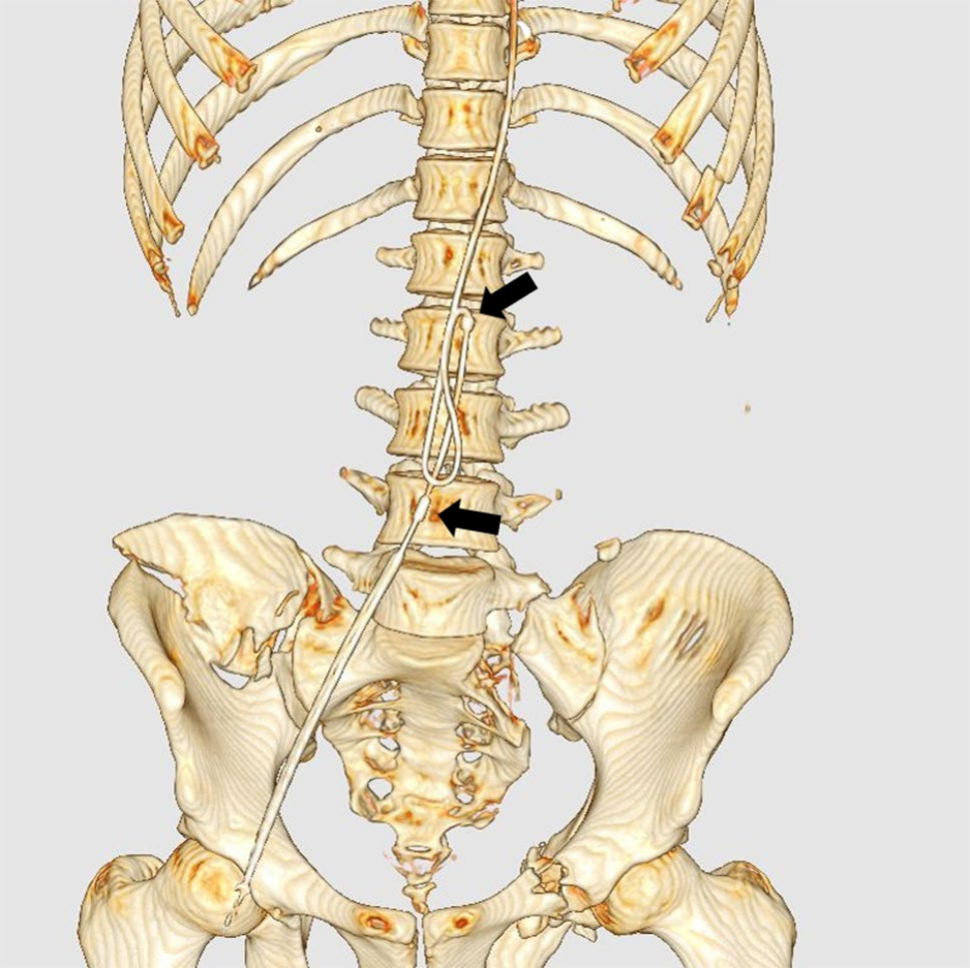
Three-dimensional computed tomography showing loop formation of the guidewire. The balloon catheter could not be inserted due to the looping of the guidewire. The black arrows indicate the radiopaque markers of the balloon catheter

The REBOA procedure can be performed blindly, which may cause loop formation of the guidewire (Fig.  7) or the balloon catheter shaft [13].

#### Migration

**Fig. 8:**
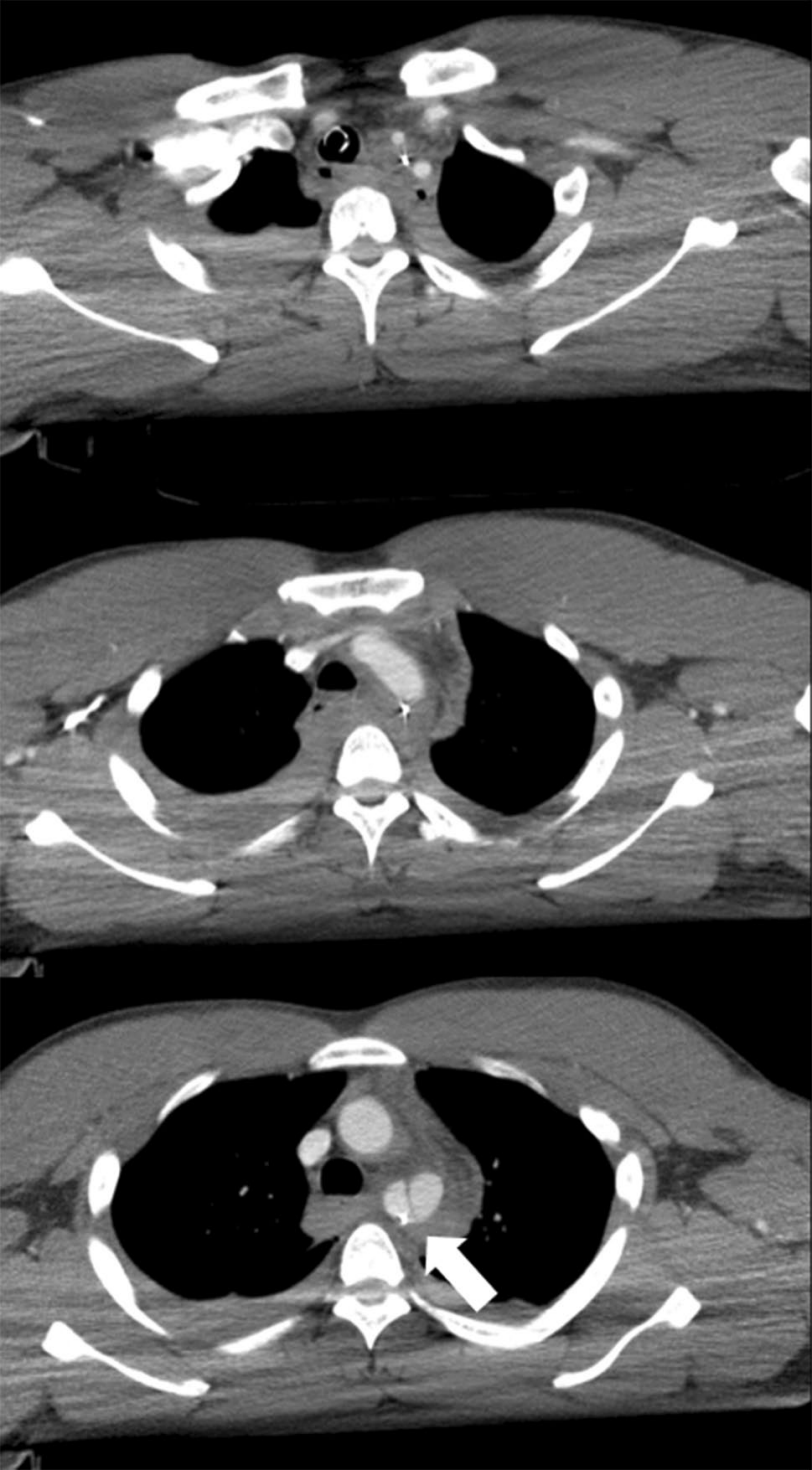
Thoracic contrast-enhancement computed tomography in a patient with multiple traumas showing a complication of the guidewire migration into the arterial subintima (arrow). Resuscitative endovascular balloon occlusion of the aorta (REBOA) is contraindicated in a thoracic aortic injury, but the injury was not expected in the REBOA procedure in this case

Unknown vessel injury or variant artery can potentially lead to device migration during the procedure (Fig.  8). Device migration may cause iatrogenic injury and render REBOA ineffective.

## Pitfalls in CT interpretation

### Hidden extravasation

**Fig. 9:**
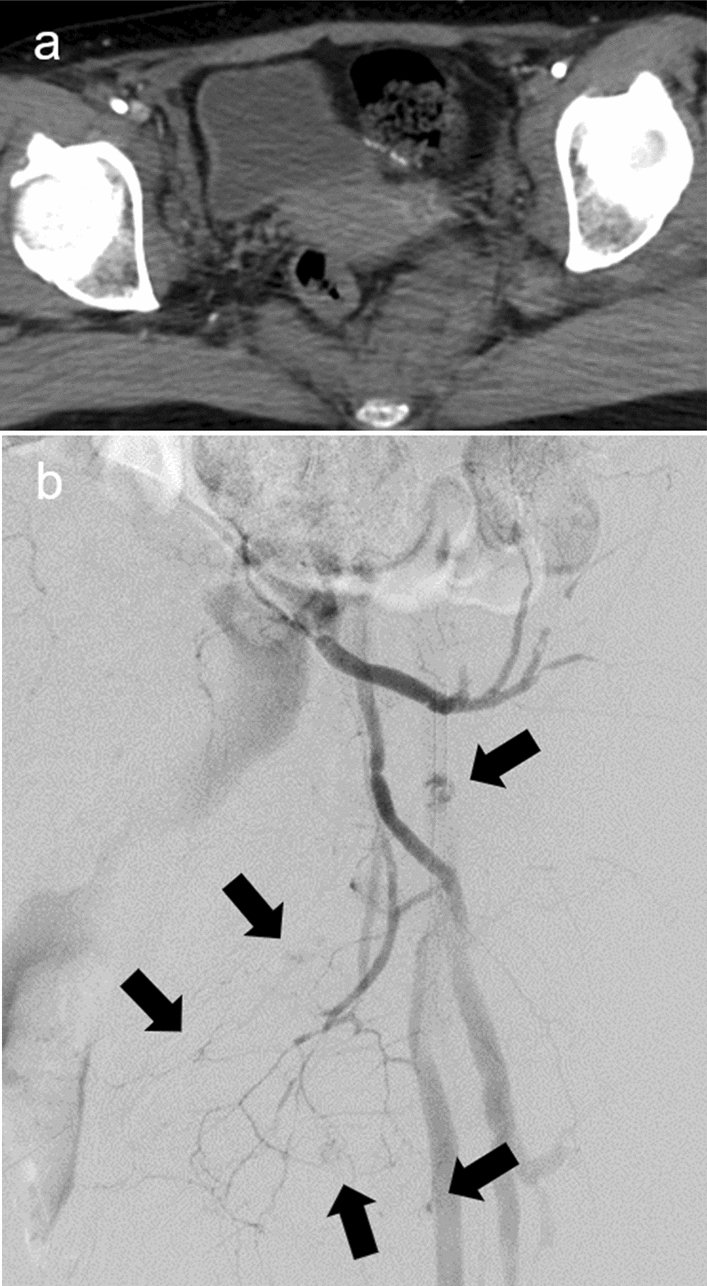
Pelvic contrast-enhancement computed tomography (CT) with complete resuscitative endovascular balloon occlusion of the aorta (REBOA) in Zone I in a patient with pelvic fractures showing a massive hematoma in the retroperitoneal space without extravasation. However, left internal iliac angiography (REBOA was deflated temporarily) shows diffuse extravasation (black arrows), which was hidden by the decreased blood flow under complete REBOA during CT scanning

Although REBOA can control subdiaphragmatic hemorrhage, hidden extravasation of the contrast medium may occur (Fig.  9). The absence of extravasation on contrast-enhanced CT for REBOA cannot rule out downstream bleeding.

### Differentiation of organ injury and ischemia

**Fig. 10:**
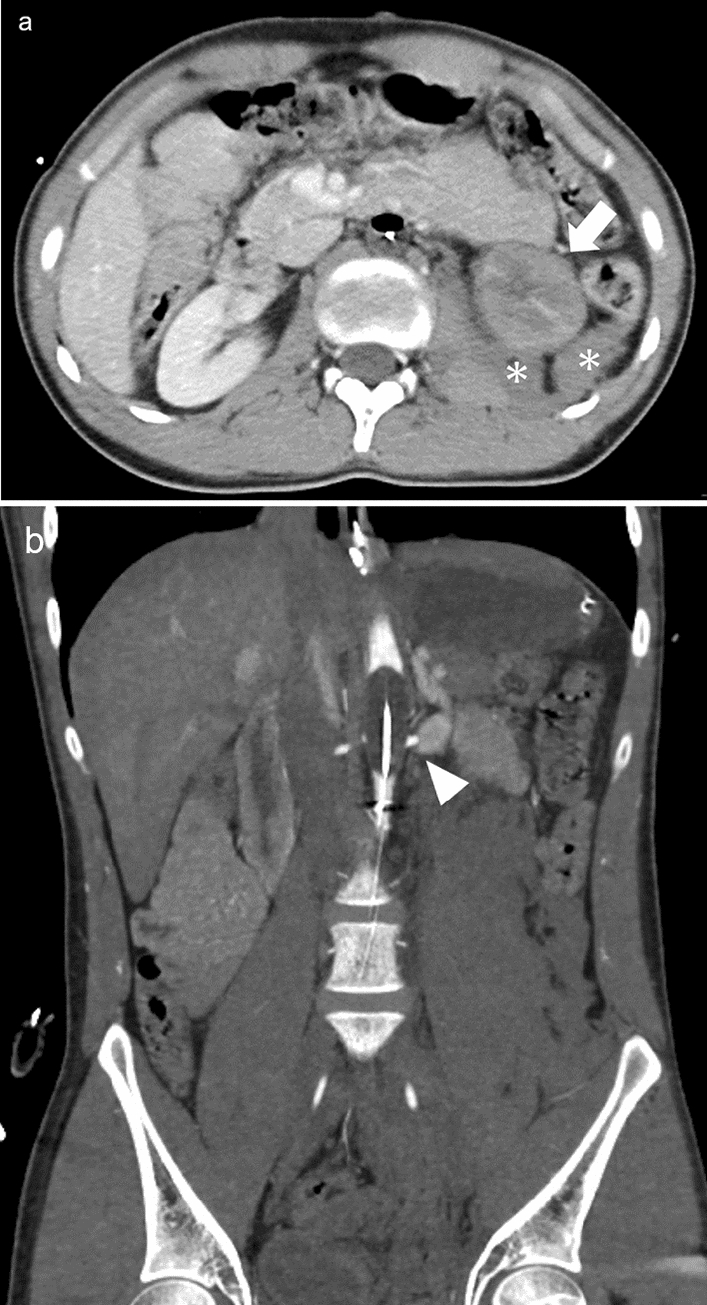
Abdominal contrast-enhancement computed tomography (**a**: axial image, **b**: coronal view) with complete resuscitative endovascular balloon occlusion of the aorta (REBOA) in a patient with multiple traumas showing decreased enhancement on the left kidney (**a**, arrow) and hematoma around it (**a**, asterisks), which is suggestive of renal contusion. However, in this case, REBOA was misplaced in Zone II and the orifice of the left renal artery was occluded by the balloon (**b**, arrowhead). Therefore, it is difficult to conclude that this case was REBOA-related hypoperfusion or renal injuries

**Fig. 11:**
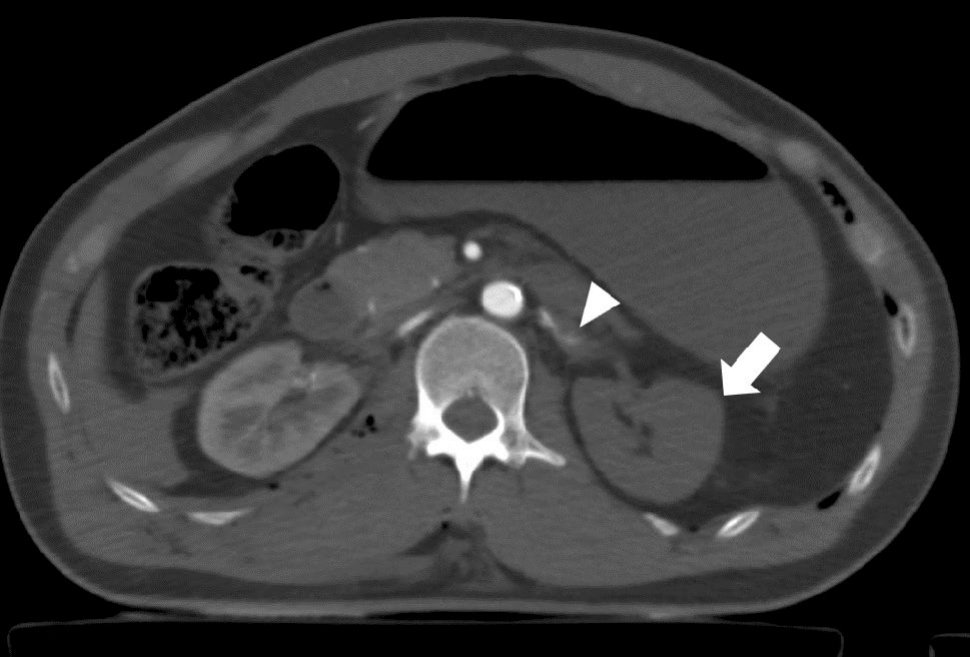
Abdominal contrast-enhancement computed tomography with partial resuscitative endovascular balloon occlusion of the aorta in Zone I in a patient with multiple traumas showing no enhancement in the left kidney (arrow). The left renal artery was irregular and occluded (vascular injury) (arrowhead)

Renal contusions or renal vascular injury are characterized by decreased enhancement of the renal parenchyma [14]. Renal injury sometimes mimics the renal ischemia caused by REBOA (Figs. 10, 11). The presence of injury around the kidney may be a clue in distinguishing between renal injury and ischemia.

### Presence of baseline aortic disease

**Fig. 12:**
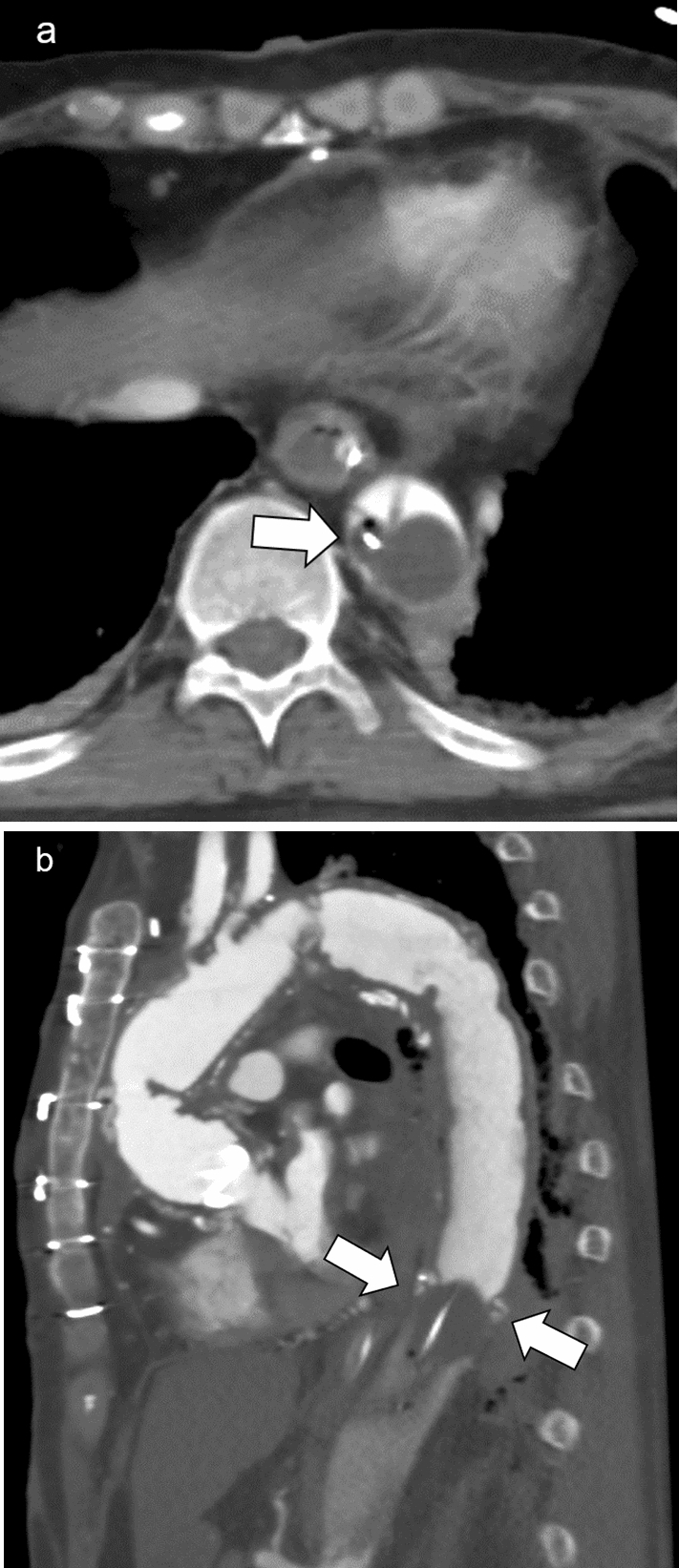
Thoracic contrast-enhancement computed tomography (**a**: axial image,** b**: sagittal view) in a patient with prior aortic replacement for aortic dissection (Stanford type A) showing that the catheter shaft was positioned in the smaller lumen (true lumen) (**a**, arrow) and the balloon was inflated in the false lumen through the double-barreled anastomosis (**b**, arrows)

The medical history may be uncertain in some emergency patients. A history of aortic diseases (e.g., aortic aneurysm, aortic dissection, and postoperative state) may cause complications or render REBOA ineffective (Fig.  12).

### Venous injuries

**Fig. 13:**
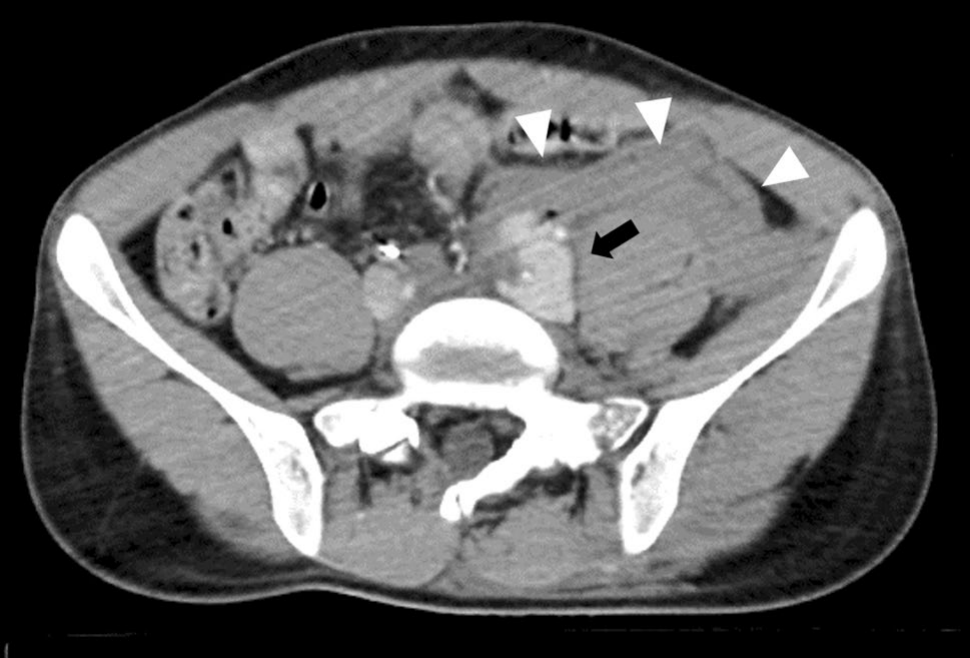
Pelvic contrast-enhancement computed tomography with complete resuscitative endovascular balloon occlusion of the aorta in a patient with multiple trauma showing massive extravasation from the left external iliac vein (black arrow) with a massive hematoma (white arrowheads)

The efficacy of REBOA in the presence of a major venous abdominal injury is unclear. REBOA appears to be effective for central venous injuries in a porcine model [15]; however, the utility of REBOA for venous injuries in humans with multiple traumatic injuries is unknown. Radiologists should highlight venous injuries to develop an appropriate treatment strategy (Fig.  13).

## Conclusion

REBOA plays an important role in patients with severe hemorrhagic shock. Accurate and prompt interpretation of CT findings is essential for treatment and can be a life-saving maneuver.
